# Laparoscopic versus open repair for peptic ulcer perforation: a systematic review, meta-analysis and trial sequential analysis of randomised controlled trials. Time to conclude!

**DOI:** 10.1308/rcsann.2024.0082

**Published:** 2024-10-03

**Authors:** BS Sokhal, AYY Mohamedahmed, S Zaman, AA Wuheb, HE Abdalla, N Husain, S Hajibandeh, S Hajibandeh

**Affiliations:** ^1^ Keele University School of Medicine, UK; ^2^ University Hospitals of North Midlands NHS Trust, UK; ^3^ University Hospitals of Derby and Burton NHS Foundation Trust, UK; ^4^ The Dudley Group NHS Foundation Trust, UK; ^5^ Wirral University Teaching Hospital NHS Foundation Trust, UK; ^6^ The Royal Wolverhampton NHS Trust, UK; ^7^ Swansea Bay University Health Board, UK

**Keywords:** Meta-analysis, Peptic ulcer disease, Surgery, Outcomes

## Abstract

**Introduction:**

The aim of this study was to investigate comparative outcomes of laparoscopic and open repair for peptic ulcer perforation (PUP).

**Methods:**

A PRISMA-compliant systematic review with a PROSPERO-registered protocol (registration number CRD42024529286) was conducted. All randomised controlled trials (RCTs) involving PUP patients managed by laparoscopic or open repair were identified and their risk of bias assessed. Outcome syntheses for perioperative mortality and morbidities, need for reoperation, procedure time and length of hospital stay were conducted using random-effects modelling to calculate risk ratios (RR) or mean difference (MD) with 95% confidence intervals (CI).

**Findings:**

Nine RCTs met the inclusion criteria, enrolling 670 patients of whom 317 were randomised to receive laparoscopic surgery and 353 were managed with open surgery. Laparoscopic repair of PUP significantly reduced mortality (RR 0.37, *p* = 0.03), total complications (RR 0.57, *p* = 0.0009), ileus (RR 0.43, *p* = 0.04), wound complications (RR 0.36, *p *< 0.0001) and length of hospital stay (MD −2.37, *p* = 0.0003) compared with the open approach. There were no significant differences in rate of postoperative leak (RR 2.00, 95% CI 0.74–5.41, *p* = 0.17), abdominal collection (RR 1.19, 95% CI 0.46–3.07, *p* = 0.72), sepsis (RR 1.17, 95% CI 0.39–3.52, *p* = 0.65), respiratory complications (RR 0.68, 95% CI 0.32–1.46, *p* = 0.32), reoperation (RR 1.74, 95% CI 0.57–5.30, *p* = 0.33) and operating time (MD 15.31, 95% CI −4.86 to 35.47, *p* = 0.14) between the two groups.

**Conclusions:**

Laparoscopic repair of PUP is associated with significantly lower mortality and morbidity and shorter length of stay compared with the open approach. The laparoscopic approach should be the management of choice subject to the existence of laparoscopic expertise.

## Introduction

Peptic ulcer disease is estimated to occur in 5%–15% of the population.^1^ Prevalence and recurrent rates of ulcers have improved in the years since the discovery and treatment of *Helicobacter pylori* in 2005.^2^ A complication of peptic ulcers is perforation, which occurs in 2%–10% of patients with peptic ulcer disease.^1,3^ Peptic ulcer perforation (PUP) can exist concurrently with sepsis in up to 35% of patients undergoing surgical management, where sepsis is the cause of death in up to 50% of cases.^4^ Therefore, perforation is a common and devastating surgical emergency.^3^

There are two common approaches for the surgical management of PUP: open or laparoscopic repair.^5^ The open abdominal approach is traditional, yet carries the risk of increased intraoperative blood loss, length of stay (LOS), postoperative pain and overall complication rates in comparison with laparoscopic surgery for a variety of emergency and elective surgical procedures.^6–8^ However, laparoscopic surgery is associated with increased procedure time and can be considered more challenging.^7^ Furthermore, use of laparoscopic surgery in the emergency setting, in the context of perforation, is inconsistent.^9^

Therefore, we aimed to conduct a meta-analysis of randomised evidence to evaluate the impact of laparoscopic vs open repair of PUP. Moreover, we planned to conduct a trial sequential analysis to assess the conclusiveness of the meta-analysis findings.

## Methods

This meta-analysis was reported in accordance with the Preferred Reporting Items for Systematic Reviews and Meta-analyses (PRISMA) statement and registered on PROSPERO (registration number CRD42024529286).^10^ This study is exempt from ethical approval because this is a secondary analysis of published data.

### Inclusion and exclusion criteria

Only randomised controlled trials (RCTs) were eligible to be included in the meta-analysis. The exclusion criteria included meta-analyses, systematic reviews, observational studies, case-control studies, case series, case reports, review articles, editorials and research letters. The review population of interest were adults (>18 years) of any sex undergoing surgery for perforated peptic ulcers only. The comparison was surgical management using open surgery vs laparoscopic surgery. The primary outcomes of interest were the adverse outcomes of mortality and complications (total complications, ileus, repair-site leak, abdominal collection, sepsis, respiratory complications, wound complications). Secondary outcomes were readmissions, LOS, operating time and reoperation.

### Screening and data extraction

Titles and abstracts of the included studies were screened by two authors. Disputes were resolved by a third reviewer. Data were extracted by two authors independently into preformed tables. Tables included study characteristics (author, publication year, study design and journal), demographic information of patients and outcome measures (total complications, ileus, repair-site leak, abdominal collection, sepsis, respiratory complications, wound complications). Any disagreements were resolved by a third reviewer.

### Risk of bias assessment

Using Cochrane’s tool for RCTs, two authors independently assessed the methodological quality of each study.^11^ The tool appraises the robustness of included studies by considering the risk of performance, reporting, detection, attrition and any other sources of bias. Disputes between authors were resolved by a third reviewer.

### Data analysis

All analyses were performed using Review Manager 5.4 with random-effects modelling.^12^ One author extracted and entered data into Review Manager 5.4. The data were cross-checked by another author. Risk ratios (RR) were calculated for dichotomous outcome measures and mean difference (MD) between groups were calculated for continuous outcome measures. Extracted data were converted to mean and standard deviation (sd) using Hozo *et al*’s equation for instances where median and interquartile range were reported instead of the mean and sd.^13^ Outcomes were reported in forest plots with 95% confidence intervals (CI), where the unit of analysis was the individual patient.

The heterogeneity among the included studies was assessed with Cochran *Q* test, with *I*^2^ calculated to assess the degree of heterogeneity. Low heterogeneity was defined as 0% to 25%, moderate heterogeneity was defined as between 25% and 75% and significant heterogeneity was defined as between 75% and 100%. Trial sequential analysis was performed to investigate the likelihood of type 1 and type 2 errors in the analyses of mortality, total complications, length of hospital stay, and operating time using random-effects modelling and a 95% CI using TSA software.^14^ To determine the likelihood of type 1 and type 2 errors, O’Brien-Fleming α-spending function and futility boundaries were used.

### Sensitivity analyses

Sensitivity analyses were conducted to assess heterogeneity and the validity of results by repeating the analysis after excluding one study at a time (leave-one-out sensitivity analysis).

## Findings

**Figure 1:**
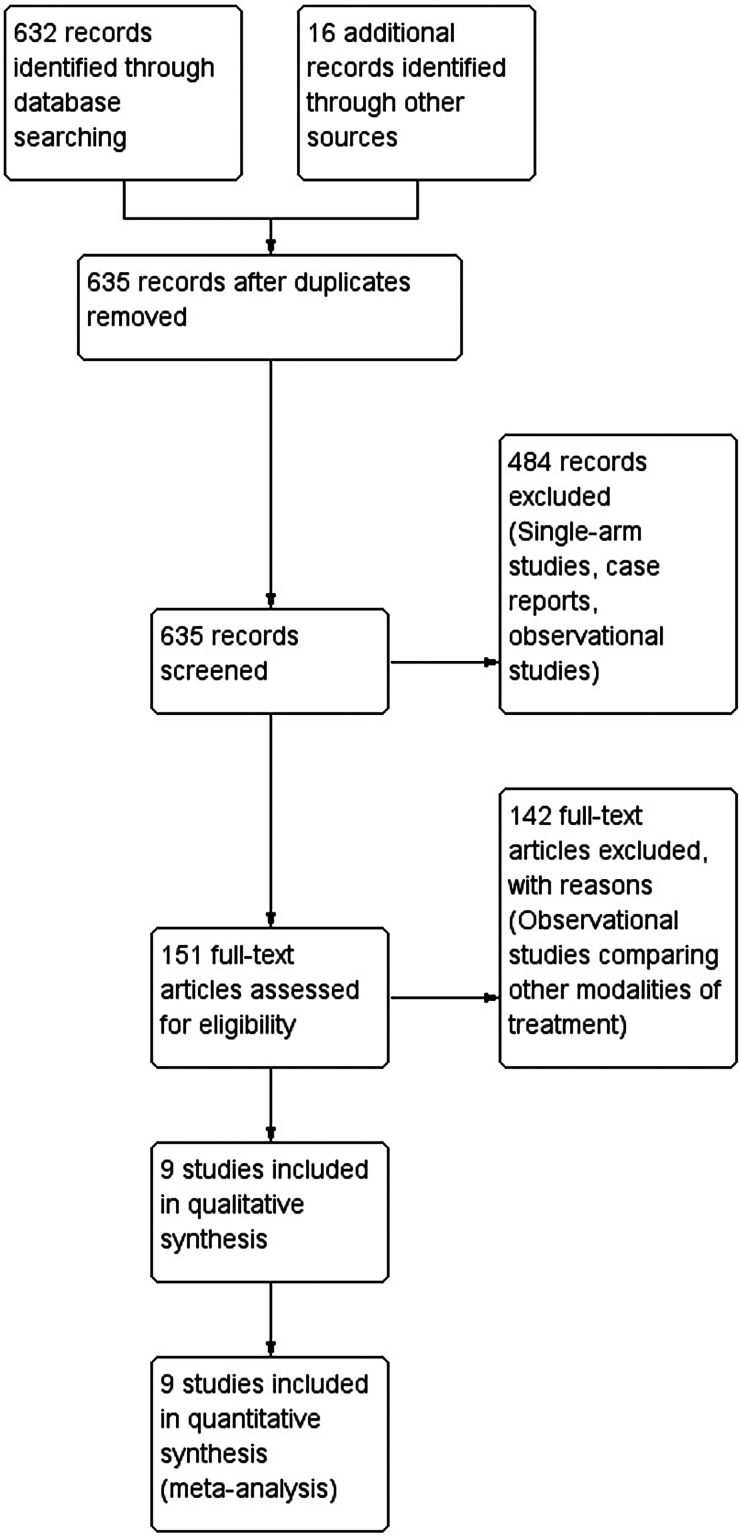
PRISMA flow chart

In total, 648 citations were retrieved, and 635 were screened after de-duplication (Figure 1). Following screening, 9 RCTs enrolling 670 patients met the inclusion criteria. One study^16^ included the number of patients converted from laparoscopic to open surgery (Figure 1). Of the 670 patients, 317 were randomised to receive laparoscopic surgery, and 353 were managed with open surgery.

Table 1 presents the year of publication, country of origin, study design, number of patients in the laparoscopic and open groups, inclusion criteria, site of perforation and size of perforation for the included studies. Table 2 outlines the baseline characteristics of the included populations including age, sex, body mass index and past medical history.

**Table 1:** Baseline characteristics of included studies

Study	Country	Type of the study	Number of patients	Inclusion and exclusion criteria	Site of perforation			Size of perforation (mm)
					Pre-pyloric	Post-pyloric	Pyloric\juxta /gastric	
Lau *et al* (1996)^9^	China	RCT	Laparoscopic 24 Open 21	Exclusion criteria: complicated ulcers that required definitive ulcer surgery; associated bleeding ulcers; unsuitability for laparoscopic procedures like previous operations; serious associated cardiopulmonary diseases that precluded a long operation; no consent from patient for randomisation; and clinical sealed-off perforated ulcers	Laparoscopic 1 Open 3	Laparoscopic 20 Open 16	Laparoscopic 3 Open 2	Laparoscopic 6 (1–20) Open 5 (2–25)
Siu *et al* (2002)^20^	China	RCT	Laparoscopic 63 Open 58	Inclusion criteria: clinical diagnosis of perforated peptic ulcer made by the surgeon and confirmed at the operating room Exclusion criteria: a history of upper abdominal surgery; concomitant evidence of bleeding from the ulcer, or gastric outlet obstruction; a surgical diagnosis other than a perforated peptic ulcer	Laparoscopic 1 Open 0	Laparoscopic 45 Open 48	Laparoscopic 17 Open 10	Laparoscopic 5.2 (4.9) Open 4.7 (3.0)
Bertleff *et al* (2009)^15^	Netherlands	RCT	Laparoscopic 52 Open 49	Exclusion criteria: inability to read the Dutch language patient information booklet; inability to complete informed consent; prior upper abdominal surgery; or current pregnancy	Laparoscopic 19 Open 22	Laparoscopic 20 Open 14	Laparoscopic 8 Open 12	Laparoscopic ∼10 Open ∼7
Zedan *et al* (2015)^21^	Egypt	RCT	Laparoscopic 21 Open 24	Inclusion criteria: patients with suspected perforated duodenal ulcer based on clinical assessment, investigation, and confirmed by exploration; either male or female of any age with Boey’s score 0 or 1 Exclusion criteria: patients with Boey’s score 2 or 3; gastric outlet obstruction; bleeding ulcer; and previous abdominal exploration	N/A	Laparoscopic 21 Open 24	N/A	Laparoscopic 5 ± 1.5 Open 5.5 ± 2.4
Ge *et al* (2016)^17^	China	RCT	Laparoscopic 58 Open 61	Inclusion criteria: consecutive adult patients with a clinical diagnosis of perforated peptic ulcer in Shanghai Tongji Hospital between January 2010 and June 2014 Exclusion criteria included: refusal to receive surgery; serious cardiopulmonary dysfunction; peptic ulcers that were simultaneously bleeding and perforated; suspected perforation of gastric cancer; and current pregnancy	Laparoscopic 19 Open 30	Laparoscopic 39 Open 31	N/A	Laparoscopic ∼5 Open ∼4
Shah *et al* (2015)^19^	India	RCT	Laparoscopic 25 Open 25	N/A	Laparoscopic 6 Open 10	Laparoscopic 3 Open 2	Laparoscopic 16 Open 13	N/A
Abdullah *et al* (2018)^16^	Egypt	RCT	Laparoscopic 33 Open 37 Note: Converted laparoscopic added to open later for analysis Laparoscopic 18 Open 52	Exclusion criteria: patients with delayed presentation (>48h); absolute contraindication for laparoscopy (uncorrectable coagulopathy, severe cardiopulmonary disease); malignant ulcers (detected by postoperative pathology); rare sites of peptic ulcer (jejunum, ileum, lower oesophagus); and other complications with perforated peptic ulcer (bleeding or stenosis)	Laparoscopic 7 Open 8	Laparoscopic 26 Open 21	Laparoscopic 0 Open 8	Laparoscopic 5.67 ± 1.67 Open 5.68 ± 2.10 Laparoscopic 5.11 ± 0.27 Open 5.87 ± 1.15
Srivastava *et al* (2018)^22^	India	RCT	Laparoscopic 31 Open 38	Inclusion criteria: patients willing to participate in the study (by taking informed consent); patients older than 16 years with a perforated peptic ulcer presenting within 24h of symptoms Exclusion criteria: patients with a surgical diagnosis other than perforated peptic ulcer; patients presenting with perforated peptic ulcer with symptoms persisting beyond 24h; patients who absconded or left the study or died during the period of study; and patients who had to be converted from laparoscopic surgery to open surgery	N/A	N/A	N/A	N/A
Saleem *et al* (2023)^18^	Egypt	RCT	Laparoscopic 25 Open 25	Inclusion criteria: patients who agreed to participate in the study (by taking informed consent); patients with perforated peptic ulcer based on clinical assessment, investigations and confirmed by exploration; either male or female of any age Exclusion criteria: patients with a surgical diagnosis other than a perforated peptic ulcer; patients with gastric outlet obstruction; bleeding ulcer; previous abdominal exploration that results in upper abdominal scare, e.g. midline, paramedian, transverse epigastric incisions; patients who absconded or left the study or died during the period of study; and patients with cardiac and chest conditions (excluded from laparoscopic)	N/A	N/A	N/A	N/A

N/A = Not available

**Table 2:** Demographic characteristics of the population

Study	Randomisation	Age mean ± sd or median (range)	Sex male: female	BMI mean ± sd or median (range)	History of pepticulcer/treatment	*Helicobacter pylori* infection	NSAID use *n* (%)	Alcohol use *n* (%)	Smoking *n* (%)
Lau *et al* (1996)^9^	Laparoscopic (24)	52.3 ± 13.8	20:4	N/A	N/A	N/A	N/A	N/A	N/A
	Open (21)	51.1 ± 19.7	17:4						
Lau *et al* (1996)^9^	Laparoscopic (24)	47.8 ± 17.5	22:2	N/A	N/A	N/A	N/A	N/A	N/A
	Open (24)	44.9 ± 18.8	20:4						
Siu *et al* (2002)^20^	Laparoscopic (63)	53.8 ± 18.4	53:10	N/A	11 (17.5)	N/A	14 (22.2)	16 (25.4)	48 (76.2)
	Open (58)	56.1 ± 19.0	45:13		15 (25.9)		12 (20.7)	16 (27.6)	42 (72.4)
Bertleff *et al* (2009)^15^	Laparoscopic (52)	66 ± 25.8	29:23	23	N/A	N/A	N/A	N/A	N/A
	Open (49)	59 ± 29.5	32:17	22					
Zedan *et al* (2015)^21^	Laparoscopic (21)	40 ± 9.4	14:7	N/A	6 (28.6)	19 (90.5)	8 (38.1)	N/A	N/A
	Open (24)	42 ± 13.4	18:6		6 (25.0)	22 (91.7)	10 (41.7)		
Ge *et al* (2016)^17^	Laparoscopic (58)	46.4 ± 20.4	49:9	N/A	18 (31.0)	N/A	N/A	4 (6.9)	15 (25.9)
	Open (61)	46.5 ± 18.0	54:7		25 (41.0)			7 (11.5)	21 (34.4)
Shah *et al* (2015)^19^	Laparoscopic (25)	50 (25–60)	20:5	N/A	12 (48.0)	N/A	N/A	N/A	N/A
	Open (25)	51 (27–62)	21:4		11 (44.0)				
Abdullah *et al* (2018)^16^	Laparoscopic (33)	46.97 ± 14.78	28:5	N/A	7 (21.2)	N/A	8 (24.2)	N/A	N/A
	Open (37)	49.92 ± 12.82	31:6		6 (16.2%)		8 (21.6)		
Saleem *et al* (2023)^18^	Laparoscopic (25) Open (25)	47.22 ± 12.52	41:9	N/A	6 (24.0)	8 (32.0)	9 (36.0)	2 (8.0)	11 (44.0)
					5 (20.0)	7 (28.0)	10 (40.0)	1 (4.0)	12 (48.0)

N/A = not available; NSAID = non-steroidal anti-inflammatory drug

### Risk of bias assessment

**Figure 2:**
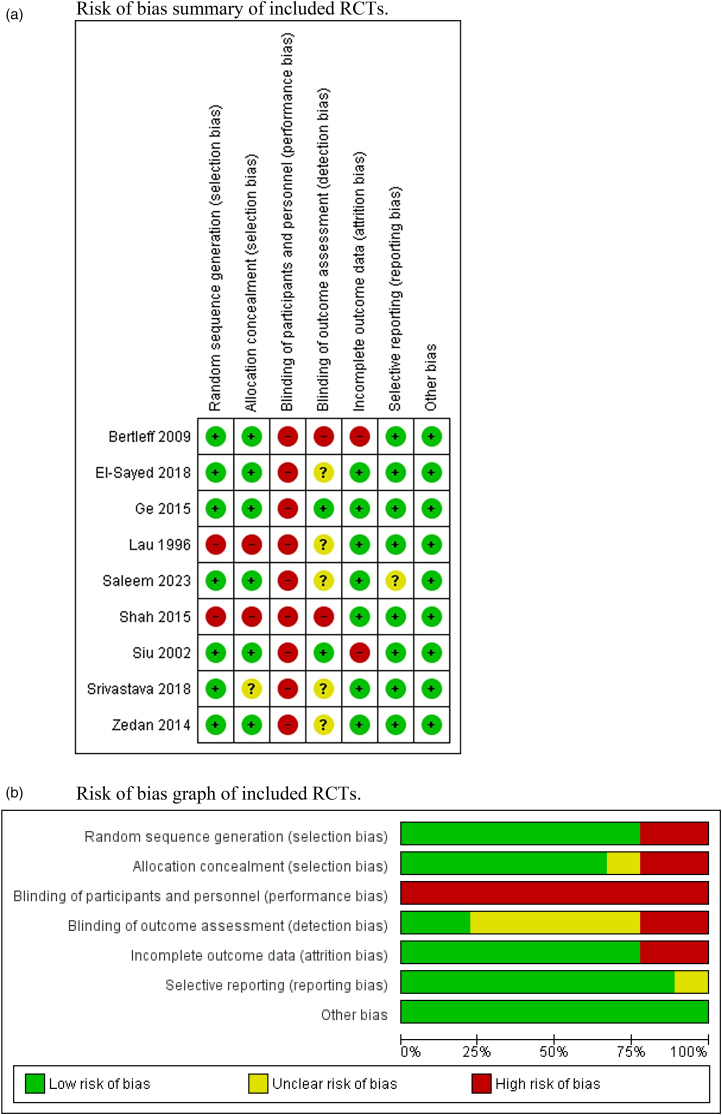
Risk of bias assessment of included randomised controlled trials

Figure 2 presents the risk of bias assessment of the included RCTs. Seven studies had a low risk of selection bias, and two studies had a high risk of selection bias. All included studies had high risk of performance bias due to being unable to blind patients, whereas the risk of detection bias was low in two studies, unclear in five studies and high in the remaining two studies. The risk of attrition bias was low in seven studies and high in two studies. The risk of reporting bias was low in eight studies and unclear in one study. The risk of other types of bias was low in seven studies and unclear in two studies.

### Outcome synthesis

**Figure 3:**
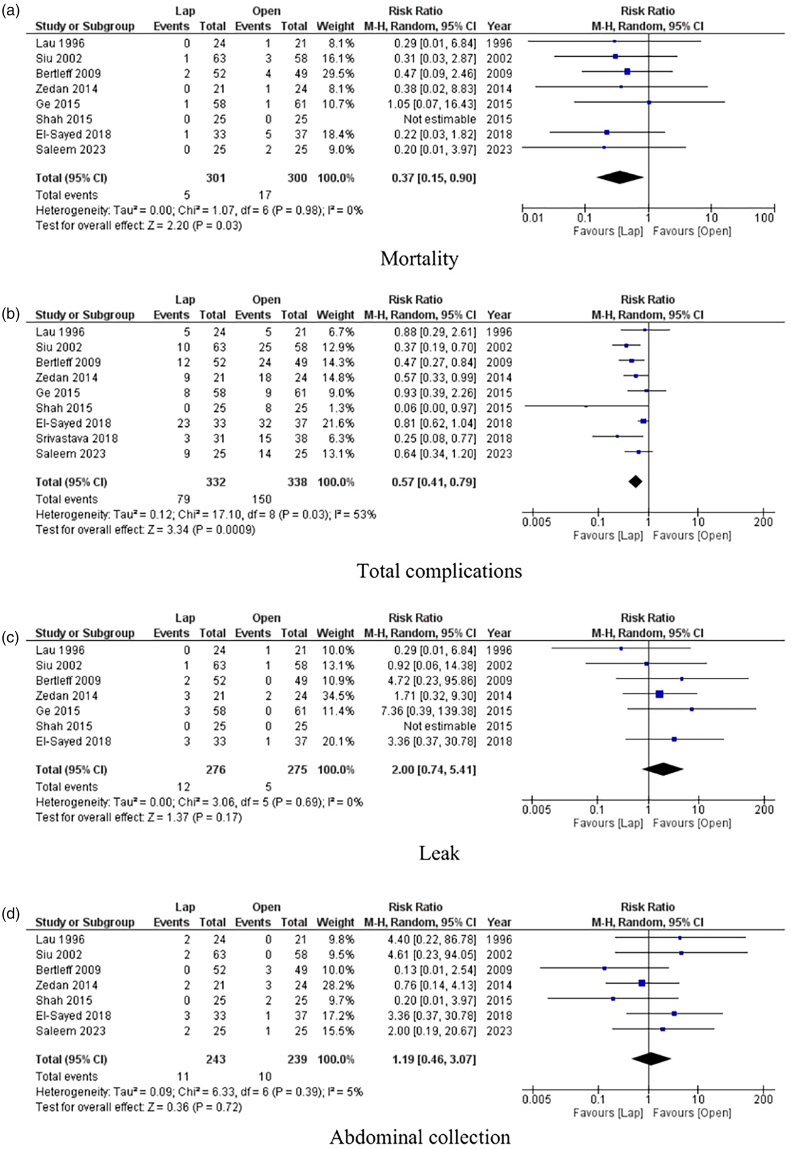

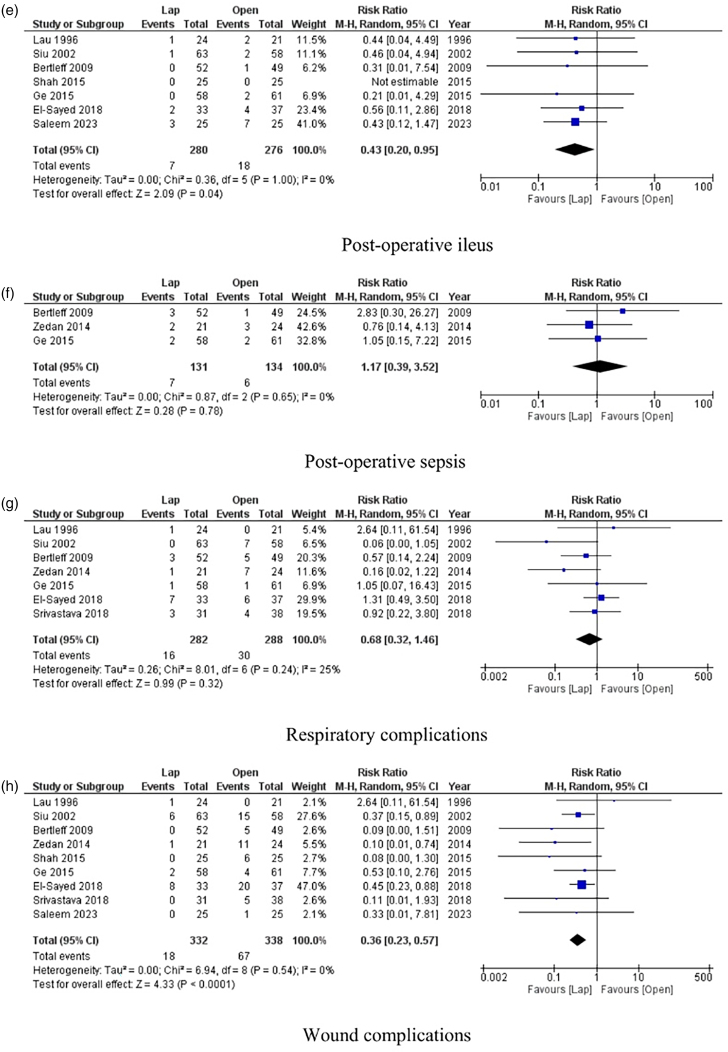

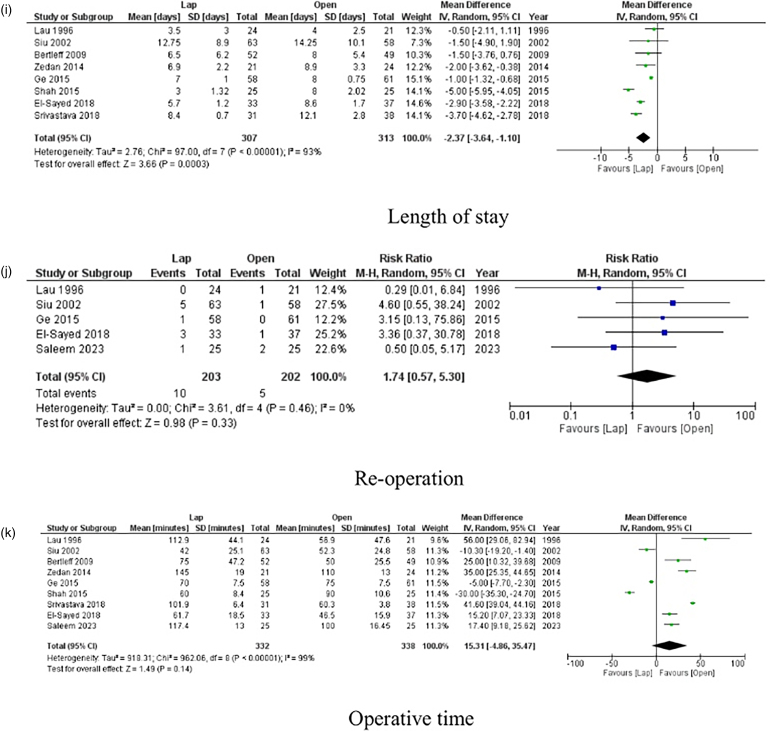
Forest plots of comparison of (a) mortality, (b) total complications, (c) leak, (d) abdominal collection, (e) postoperative ileus, (f) postoperative sepsis, (g) respiratory complications, (h) wound complications, (i) length of stay, (j) reoperation and (k) operating time. Solid squares denote the odds ratio or risk difference. Horizontal lines represent 95% confidence intervals and the diamond denotes the pooled effect size. M–H = Mantel Haenszel test

Outcomes are summarised in Figure 3.

#### Mortality

Eight studies reported mortality as an outcome.^9,15–21^ The mortality rate was 1.7% in the laparoscopic surgery group and 5.7% in the open surgery group. Laparoscopic surgery was associated with a significantly lower mortality rate compared with open surgery (RR 0.37, 95% CI 0.15–0.90, *p* = 0.03). There was low between-study heterogeneity (*I*^2^ = 0%, *p* = 0.98).

#### Total complications

Nine studies reported total complications as an outcome.^9,15–22^ The rate of total complications was 24.8% in the laparoscopic surgery group and 44.4% in the open surgery group. Laparoscopic surgery was associated with a significantly lower rate of total complications compared with open surgery (RR 0.57, 95% CI 0.41–0.79, *p* = 0.0009). There was moderate between-study heterogeneity (*I*^2^ = 53%, *p* = 0.03).

#### Postoperative leak

Seven studies reported leak from repaired PUP as an outcome.^9,15,17–21^ The rate of leak was 4.3% in the laparoscopic surgery group and 1.8% in the open surgery group. There was no significant difference between the laparoscopic surgery and open surgery groups (RR 2.00, 95% CI 0.74–5.41, *p* = 0.17). There was low between-study heterogeneity (*I*^2^ = 0%, *p* = 0.69).

#### Abdominal collection

Seven studies reported abdominal collection as an outcome.^9,15,17–21^ The rate of abdominal collection was 4.5% in the laparoscopic surgery group and 4.2% in the open surgery group. There was no significant difference between the laparoscopic surgery and open surgery groups (RR 1.19, 95% CI 0.46–3.07, *R* = 0.72). There was low between-study heterogeneity (*I*^2^ = 0%, *p* = 0.39).

#### Postoperative ileus

Seven studies reported postoperative ileus as an outcome.^9,15,17–21^ The rate of postoperative ileus was 2.5% in the laparoscopic surgery group and 6.5% in the open surgery group. Laparoscopic surgery was associated with a significantly lower rate of postoperative ileus compared with open surgery (RR 0.43, 95% CI 0.20–0.95, *p* = 0.04). There was low between-study heterogeneity (*I*^2^ = 0%, *p* = 1.00).

#### Sepsis

Three studies reported postoperative sepsis as an outcome.^15,17,21^ The rate of sepsis was 5.3% in the laparoscopic surgery group and 4.5% in the open surgery group. There was no significant difference between the laparoscopic surgery and open surgery groups (RR 1.17, 95% CI 0.39–3.52, *p* = 0.65). There was low between-study heterogeneity (*I*^2^ = 0%, *p* = 0.78).

#### Respiratory complications

Seven studies reported respiratory complications as an outcome.^9,15,17,19–22^ The rate of respiratory complications was 5.7% in the laparoscopic surgery group and 10.4% in the open surgery group. There was no significant difference between the laparoscopic surgery and open surgery groups (RR 0.68, 95% CI 0.32–1.46, *p* = 0.32). There was low between-study heterogeneity (*I*^2^ = 25%, *p* = 0.24).

#### Wound complications

Nine studies reported wound complications as an outcome.^9,15–22^ The rate of wound complications was 5.4% in the laparoscopic surgery group and 19.8% in the open surgery group. Laparoscopic surgery was associated with a significantly lower rate of wound complications compared with open surgery (RR 0.36, 95% CI 0.23–0.57, *p *< 0.0001). There was low between-study heterogeneity (*I*^2^ = 0%, *p* = 0.54).

#### Length of hospital stay

Eight studies reported the LOS of their patients as an outcome.^9,15,17–22^ Laparoscopic surgery was associated with significantly lower LOS compared with open surgery (MD −2.37, 95% CI: −3.64 to −1.10, *p* = 0.0003). There was high between-study heterogeneity (*I*^2^ = 93%, *p *< 0.00001).

#### Reoperation

Five studies reported reoperation rate as an outcome. The reoperation rate was 4.9% in the laparoscopic surgery group and 2.5% in the open surgery group. There was no significant difference between the laparoscopic surgery and open surgery groups (RR 1.74, 95% CI 0.57–5.30, *p* = 0.33). There was moderate between-study heterogeneity (*I*^2^ = 46%, *p* = 0.46).

#### Operative time

Nine studies reported the operating time as an outcome.^9,15–22^ There was no significant difference between the laparoscopic surgery and open surgery groups (MD 15.31, 95% CI −4.86 to 35.47, *p* = 0.14). There was high between-study heterogeneity (*I*^2^ = 99%, *p*<0.00001).

### Sensitivity analysis

The direction of pooled effect size remained unchanged when the OR or risk difference was calculated. Furthermore, the leave-one-out analysis has not demonstrated important discrepancies with the original analysis.

### Trial sequential analysis

**Figure 4:**
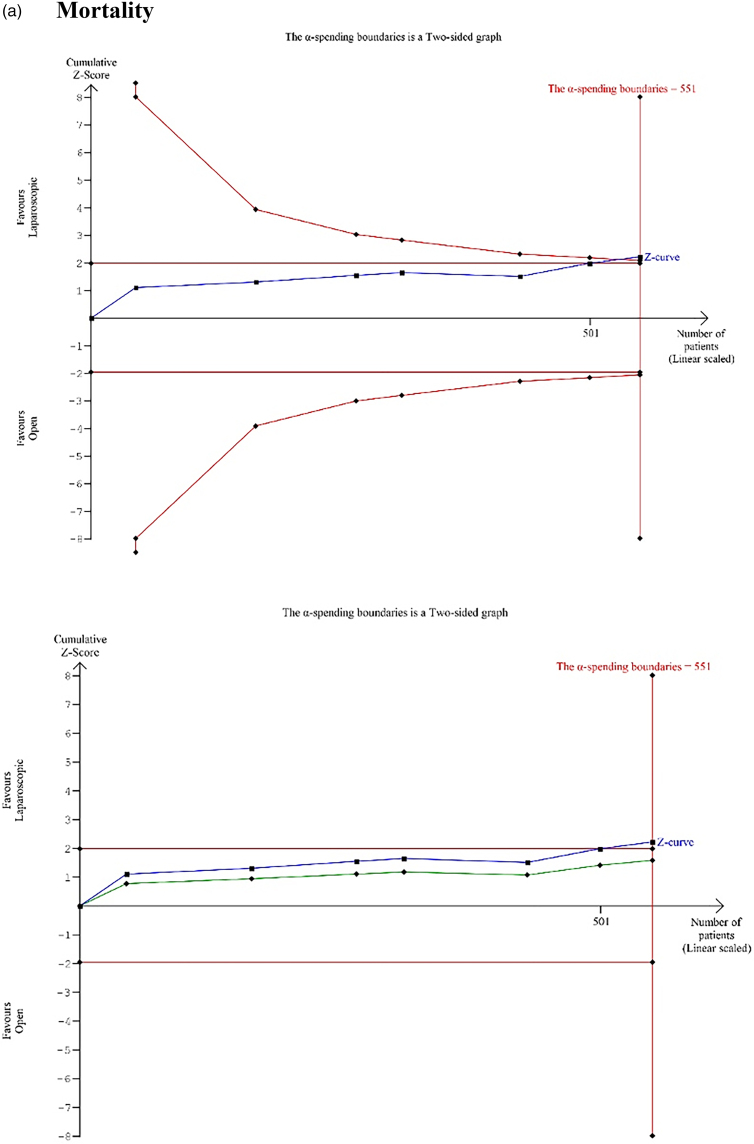

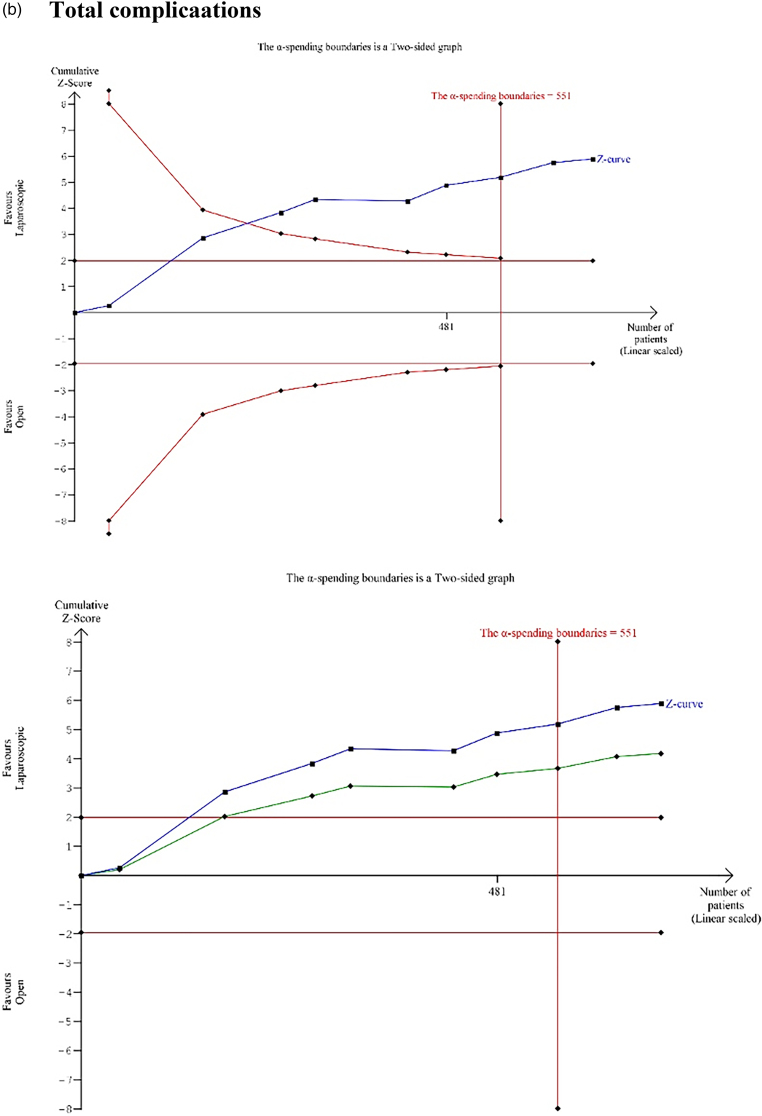

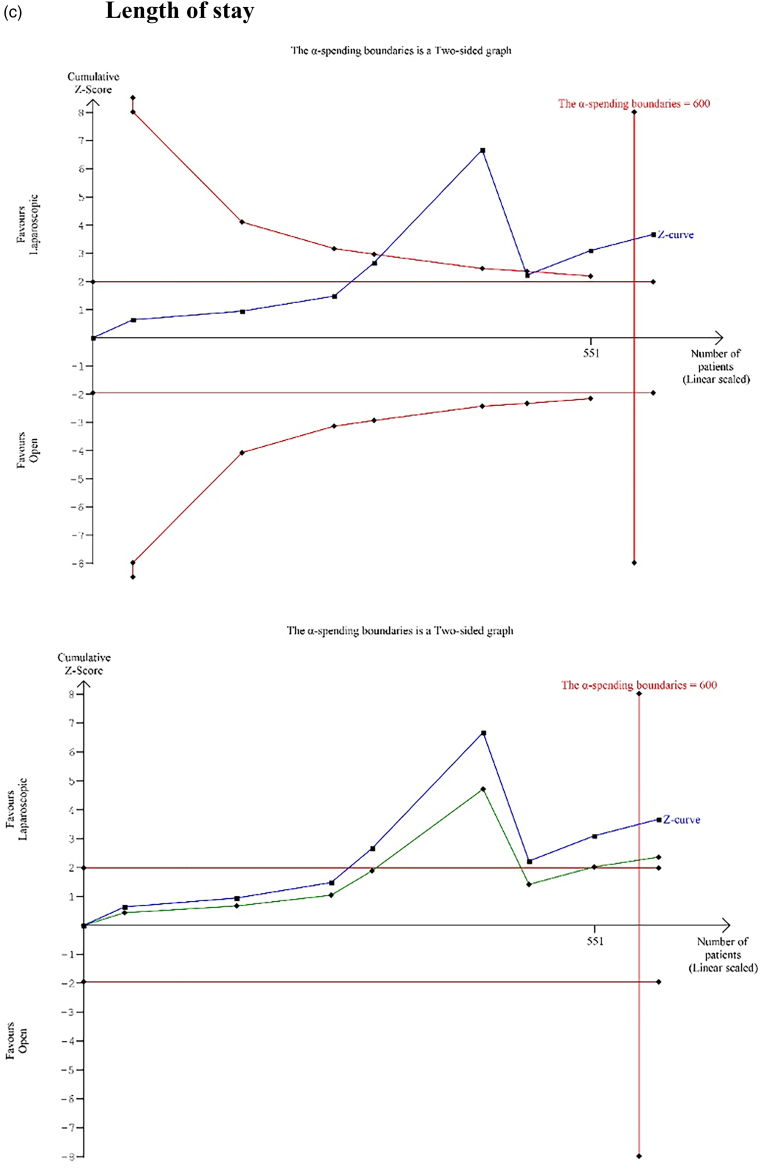

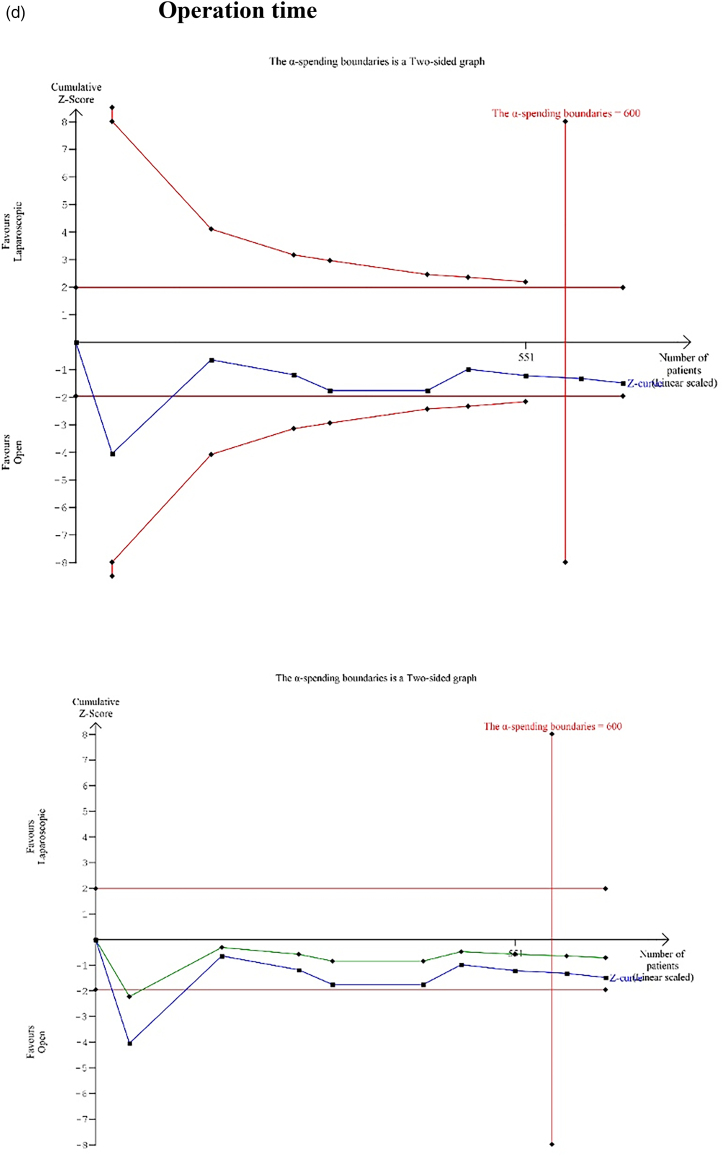
Results of trial sequential analysis for (a) mortality, (b) total complications, (c) length of stay and (d) operating time. The red inward-sloping dashed lines make up the trial sequential monitoring boundaries. To the right, the outward sloping red dashed lines make up the futility region. The solid blue line is the cumulative *Z*-curve. The solid green line is the penalised *Z*-value.

The outcomes of trials sequential analysis are presented in Figure 4.

#### Mortality

The calculated information size was 551 patients. The *Z*-curve crossed the conventional boundaries and alpha-spending boundaries in favour of the laparoscopic approach before and after the information size was reached and the penalised *Z*-value remained >1.96; therefore, the meta-analysis was conclusive, and the risk of type 1 error was minimal.

#### Total complications

The calculated information size was 551 patients. The *Z*-curve crossed the conventional boundaries and alpha-spending boundaries in favour of the laparoscopic approach before and after the information size was reached and the penalised *Z*-value remained >1.96; therefore, the meta-analysis was conclusive, and the risk of type 1 error was minimal.

#### Length of hospital stay

The calculated information size was 600 patients. The *Z*-curve crossed the conventional boundaries and alpha-spending boundaries in favour of the laparoscopic approach before and after the information size was reached and the penalised *Z*-value remained >1.96; therefore, the meta-analysis was conclusive, and the risk of type 1 error was minimal.

#### Operative time

The information size was calculated at 600 patients. The *Z*-curve did not cross the conventional boundaries and the absolute number for the penalised *Z*-value remained <1.96 in both sides after the information size is reached; therefore, the meta-analysis was conclusive, and the risk of type 2 error was minimal.

## Discussion

This comprehensive meta-analysis of level 1a evidence on laparoscopic vs open repair of PUP demonstrated that laparoscopic repair of PUP significantly reduced mortality, total complications, ileus, wound complications and LOS compared with the open approach. Furthermore, there were no significant differences in rate of postoperative leak, abdominal collection, sepsis, respiratory complications, reoperation and operating time between two groups. Despite the presence of high between-study heterogeneity in the analyses of operating time and LOS, and moderate between-study heterogeneity in the analysis of total complications, most outcome syntheses demonstrated low between-study heterogeneity, suggesting robustness of these findings.

For the first time in the current literature, to evaluate the conclusiveness of our meta-analysis findings, we performed a trial sequential analysis, which confirmed that our findings are not subject to risk of type 1 or type 2 errors, indicating that the available sample sizes provided by the existing RCTs are sufficient to make a conclusion about this topic.

The optimal management for PPUs is debated. Studies have found that laparoscopic repair leads to reduced blood loss and LOS but increased operating times.^3,4,23^ However, it is important to note that these studies derive data from elective surgery and, given the increased difficulty of laparoscopic repair, it is favoured in high-volume centres with more experienced surgeons.^24^ Therefore, there is no consensus over whether laparoscopic repair is superior to open repair. This review provides high-quality evidence favouring the use of laparoscopic repair, because of favourable outcomes with lower complication rates and an insignificant difference in operating times. There have been previous meta-analyses demonstrating that laparoscopic surgery has favourable outcomes compared with open surgery for PPUs.^23^ These studies have consistently demonstrated that laparoscopic repair led to reduced morbidity and LOS.^23,25,26^ This study adds to previous evidence because of the inclusion of more trials and a trial sequential analysis to assess to the risk of residual type 1 and type 2 error. Furthermore, low heterogeneity between studies for most outcomes supports the robustness of these findings.

This study supports the use of laparoscopic repair in the emergency setting. Most studies comparing laparoscopic repair with open repair are conducted in the elective setting, where there is a clear benefit for reducing postoperative complications and LOS.^27^ Previously laparoscopic repair was thought to have increased the risk of bacteraemia in patients with perforation and peritonitis.^28^ This study adds to the evidence suggesting that laparoscopic surgery is safe in patients with increased risk of sepsis in comparison with open repair, with postoperative sepsis rates of 5.3%, and is not significantly different from the rate for open surgery. Laparoscopic repair has previously been associated with increased risk of repair-site leak requiring further surgery.^29^ Our study of pooled trial evidence showed a 4.3% rate of repair-site leak, which was not statistically significant when compared with the 1.8% rate of open surgery. Since the inception of laparoscopic surgery, it has been established as the first option for many surgeries. Improved equipment, expertise and overall uptake of laparoscopic surgery have led to superior outcomes when compared with open surgery for a variety of conditions.^30,31^ This is reflected in previous studies showing a conversion rate of more than 1 in 5, which is reduced to under 1 in 10 in this meta-analysis.^32^ Furthermore, an established limitation of laparoscopic surgery was increased operating time and costs.^33,34^ The current study found no statistically significant difference in operating times between laparoscopic and open repair of PPUs. As mentioned previously, this could be because of improved surgeon technique and advances in medical equipment. Alternatively, this could be due to a selection bias within trials where patients already at lower risk of complications from invasive procedures are more likely to be recruited, hence their treatment is likely to be less complicated when compared with the average patient, rendering outcomes less generalisable.^35^

### Clinical implications

There are important clinical implications to this study. We demonstrate that laparoscopic repair is favourable in comparison with open repair of perforation because of the lower likelihood of adverse outcomes. In addition, this shows that laparoscopic repair is safe in the context of emergency surgery where there is already an increased risk of sepsis because of the presence of perforation and peritonitis. It comes with decreased risk of morbidity, infection and LOS without the compromise of operating time. Therefore, laparoscopic surgery should be more routinely considered in patients undergoing repair for PPUs.

### Limitations

There are several limitations to this study. There was high risk of geographical publication bias. Most studies were conducted in Asia and Africa, with only one study conducted in Europe. Therefore, the results may not be generalisable to different population groups. There was high risk of inevitable performance bias because of a lack of blinding in all the included studies and high or unclear risk of detection bias in most of the included studies. Although blinding of the participants was not possible, the blinding of outcome assessors could have been tried. There was a moderate to high risk of between-study heterogeneity in some of our evaluated outcome syntheses. Finally, inherently from the research design, this study is subject to publication bias.

## Conclusions

The meta-analysis of randomised evidence (level 1a) demonstrated that laparoscopic repair of PUP is associated with significantly lower mortality and morbidity, and shorter LOS compared with the open approach. The trial sequential analysis confirmed the conclusiveness of the meta-analysis findings. The laparoscopic approach should be the management of choice for PUP depending on clinical features, surgeon experience and patient suitability.

## Authors contributions

Study conception and design: S Hajibandeh, AYY Mohamedahmed. Acquisition of data: AA Wuheb, HE Abdalla. Analysis and interpretation of data: BS Sokhal, AYY Mohamedahmed, S Hajibandeh, S Hajibandeh. Drafting of manuscript: BS Sokhal, AYY Mohamedahmed, S Hajibandeh. Critical revision of manuscript: BS Sokhal, AYY Mohamedahmed, S Zaman, AA Wuheb, HE Abdalla, N Husain, S Hajibandeh, S Hajibandeh.

## References

[1] Ocasio Quinones GA, Woolf A. Duodenal ulcer. In: *StatPearls*. Treasure Island (FL): StatPearls Publishing. Copyright © 2023. StatPearls Publishing LLC; 2023. 32491322

[2] Bordin DS, Shengelia MI, Ivanova VA, Voynovan IN. The history of the discovery of the *Helicobacter pylori*. *Ter Arkh* 2022; **94**: 283–288. 36286752 10.26442/00403660.2022.02.201377

[3] Ansari D, Torén W, Lindberg S *et al.* Diagnosis and management of duodenal perforations: a narrative review. *Scand J Gastroenterol* 2019; **54**: 939–944. 31353983 10.1080/00365521.2019.1647456

[4] Amini A, Lopez RA. Duodenal perforation. In: *StatPearls*. Treasure Island (FL): StatPearls Publishing. Copyright © 2023, StatPearls Publishing LLC; 2023. 31971724

[5] Chung KT, Shelat VG. Perforated peptic ulcer - an update. *World J Gastrointest Surg* 2017; **9**: 1–12. 28138363 10.4240/wjgs.v9.i1.1 PMC5237817

[6] Zhou MW, Gu XD, Xiang JB, Chen ZY. Clinical safety and outcomes of laparoscopic surgery versus open surgery for palliative resection of primary tumors in patients with stage IV colorectal cancer: a meta-analysis. *Surg Endosc* 2016; **30**: 1902–1910. 26281903 10.1007/s00464-015-4409-1

[7] Kiblawi R, Zoeller C, Zanini A *et al.* Laparoscopic versus open pediatric surgery: three decades of comparative studies. *Eur J Pediatr Surg* 2022; **32**: 9–25. 34933374 10.1055/s-0041-1739418

[8] Zhang Y, Liu C, Nistala KRY, Chong CS. Open versus laparoscopic Hartmann’s procedure: a systematic review and meta-analysis. *Int J Colorectal Dis* 2022; **37**: 2421–2430. 36416926 10.1007/s00384-022-04285-6

[9] Lau WY, Leung KL, Kwong KH *et al.* A randomized study comparing laparoscopic versus open repair of perforated peptic ulcer using suture or sutureless technique. *Ann Surg* 1996; **224**: 131–138. 8757375 10.1097/00000658-199608000-00004 PMC1235333

[10] Page MJ, McKenzie JE, Bossuyt PM *et al.* The PRISMA 2020 statement: an updated guideline for reporting systematic reviews. *Syst Rev* 2021; **10**: 89. 33781348 10.1186/s13643-021-01626-4 PMC8008539

[11] Higgins JP, Altman DG, Gotzsche PC *et al.* The Cochrane Collaboration’s tool for assessing risk of bias in randomised trials. *BMJ* 2011; **343**: d5928. 22008217 10.1136/bmj.d5928 PMC3196245

[12] Cumpston M, Li T, Page MJ *et al.* Updated guidance for trusted systematic reviews: a new edition of the Cochrane handbook for systematic reviews of interventions. *Cochrane Database Syst Rev* 2019; **10**: ED000142. 31643080 10.1002/14651858.ED000142 PMC10284251

[13] Hozo SP, Djulbegovic B, Hozo I. Estimating the mean and variance from the median, range, and the size of a sample. *BMC Med Res Methodol* 2005; **5**: 13. 15840177 10.1186/1471-2288-5-13 PMC1097734

[14] TSA software 0.9.5.5 Beta. Copenhagen: Copenhagen Trial Unit.

[15] Bertleff MJ, Halm JA, Bemelman WA *et al.* Randomized clinical trial of laparoscopic versus open repair of the perforated peptic ulcer: the LAMA trial. *World J Surg* 2009; **33**: 1368–1373. 19430829 10.1007/s00268-009-0054-y PMC2691927

[16] Abdullah E-S, Negm A, Samir M *et al.* Laparoscopic versus open repair of perforated peptic ulcer: comparative study. *World J Surg* 2018; **86**: 1767–1775.

[17] Ge B, Wu M, Chen Q *et al.* A prospective randomized controlled trial of laparoscopic repair versus open repair for perforated peptic ulcers. *Surgery* 2016; **159**: 451–458. 26297055 10.1016/j.surg.2015.07.021

[18] Saleem A-E-AA, Arafa MW, Galal AM. A comparative study of laparoscopic versus laparotomy repair of perforated peptic ulcer: a prospective study. *Egypt J Surg* 2023; **42**.

[19] Shah FH, Mehta SG, Gandhi MD, Saraj. Laparoscopic peptic ulcer perforation closure: the preferred choice. *Indian J Surg* 2015; **77**(Suppl 2):403–406. 10.1007/s12262-013-0853-0 PMC4692862 26730034

[20] Siu WT, Leong HT, Law BK *et al.* Laparoscopic repair for perforated peptic ulcer: a randomized controlled trial. *Ann Surg* 2002; **235**: 313–319. 11882751 10.1097/00000658-200203000-00001 PMC1422436

[21] Zedan A, Lolah M, Badr M, Ammar M. Laparoscopic versus open repair of perforated duodenal peptic ulcer: a randomized controlled trial. *Menoufia Med J* 2015; **28**: 62–68.

[22] Srivastava V, Singh G, Singh S. Laparoscopic repair of perforated peptic ulcers without omental patch versus conventional open surgery. *Int Surg J* 2018; **5**: 927–930.

[23] Tan S, Wu G, Zhuang Q *et al.* Laparoscopic versus open repair for perforated peptic ulcer: A meta analysis of randomized controlled trials. *Int J Surg* 2016; **33**(Pt A):124–132. 27504848 10.1016/j.ijsu.2016.07.077

[24] Vossler JD, Pavlosky KK, Murayama SM *et al.* Predictors of robotic versus laparoscopic inguinal hernia repair. *J Surg Res* 2019; **241**: 247–253. 31035139 10.1016/j.jss.2019.03.056

[25] Antoniou SA, Antoniou GA, Koch OO *et al.* Meta-analysis of laparoscopic versus open repair of perforated peptic ulcer. *J Soc Laparoendosc Surg* 2013; **17**: 15. 10.4293/108680812X13517013317752 PMC3662736 23743368

[26] Sanabria A, Villegas MI, Uribe CHM. Laparoscopic repair for perforated peptic ulcer disease. *Cochrane Database Syst Rev* 2013; **2**:. 10.1002/14651858.CD004778.pub3 PMC10837785 23450555

[27] Lee C-Z, Kao L-T, Lin H-C, Wei P-L. Comparison of clinical outcome between laparoscopic and open right hemicolectomy: a nationwide study. *World J Surg Oncol* 2015; **13**: 1–7. 26271770 10.1186/s12957-015-0666-7 PMC4536701

[28] Quah GS, Eslick GD, Cox MR. Laparoscopic repair for perforated peptic ulcer disease has better outcomes than open repair. *J Gastrointest Surg* 2019; **23**: 618–625. 30465190 10.1007/s11605-018-4047-8

[29] Bertleff MJ, Lange JF. Perforated peptic ulcer disease: a review of history and treatment. *Dig Surg* 2010; **27**: 161–169. 20571260 10.1159/000264653

[30] Deijen CL, Vasmel JE, de Lange-de Klerk ESM *et al.* Ten-year outcomes of a randomised trial of laparoscopic versus open surgery for colon cancer. *Surg Endosc* 2017; **31**: 2607–2615. 27734203 10.1007/s00464-016-5270-6 PMC5443846

[31] Ruiz-Tovar J, Diez-Tabernilla M, Chames A *et al.* Clinical outcome at 10 years after laparoscopic versus open Nissen fundoplication. *J Laparoendosc Adv Surg Tech A* 2010; **20**: 21–23. 19916741 10.1089/lap.2009.0230

[32] Shah PC, de Groot A, Cerfolio R *et al.* Impact of type of minimally invasive approach on open conversions across ten common procedures in different specialties. *Surg Endosc* 2022; **36**: 6067–6075. 35141775 10.1007/s00464-022-09073-5 PMC9283176

[33] Shushan A, Mohamed H, Magos AL. A case-control study to compare the variability of operating time in laparoscopic and open surgery. *Hum Reprod* 1999; **14**: 1467–1469. 10357960 10.1093/humrep/14.6.1467

[34] Hannan E, Lim E, Feeney G *et al.* Laparoscopic versus open appendicectomy performed by adult general surgeons in pre-teenage years children: a single-centre experience. *Ann R Coll Surg Engl* 2024. 10.1308/rcsann.2023.0044 PMC11658871 38362753

[35] Hempenius L, Slaets JP, Boelens MA *et al.* Inclusion of frail elderly patients in clinical trials: solutions to the problems. *J Geriatr Oncol* 2013; **4**: 26–31. 24071489 10.1016/j.jgo.2012.08.004

